# A randomized controlled trial study on effectiveness between tadalafil versus combination mirabegron and solifenacin on treatment of ureteral stent-related symptoms

**DOI:** 10.11604/pamj.2023.46.2.38100

**Published:** 2023-09-04

**Authors:** Tjia Adynata Ciayadi, Muhammad Asykar Palinrungi, Khoirul Kholis, Joko Hendarto, Syakri Syahrir, Syarif Syarif, Abdul Azis

**Affiliations:** 1 Department of Surgery, Faculty of Medicine, Hasanuddin University, Makassar, Indonesia,; 2 Division of Urology, Department of Surgery, Faculty of Medicine, Hasanuddin University, Makassar, Indonesia,; 3 Department of Public Health, Faculty of Medicine, Hasanuddin University, Makassar, Indonesia

**Keywords:** Adrenergic agonist, anti-muscarinic, double-J (DJ) stent, mirabegron, randomized controlled trial

## Abstract

**Introduction:**

ureteral stents have common complications like ureteral stent-related symptoms (SRSs). This study investigated the effectiveness of tadalafil compared to mirabegron and solifenacin combination therapy in patients with ureteral SRSs after double-J (DJ) stent insertion.

**Methods:**

this double-blind, randomized clinical trial used consecutive random sampling in participants with SRSs after double-J stent insertion. The study was conducted at four different hospitals in Makassar, Indonesia, from July to December 2020. Ureteral stent-related morbidity indices which analyzed include urinary symptoms, pain, general health, quality of work, and sex scores. All of the indices were measured by ureteral symptom score questionnaire for the first, second, third, and fourth weeks after drug consumption, either tadalafil 10 mg/day (group A, n=25) and a combination of mirabegron 25 mg/day and solifenacin 5 mg/day (group B, n=28).

**Results:**

before the treatment procedure, the groups were comparable in age, gender, body mass index, DJ stent procedures, type, and indication. In general, the score in all parameters declined over the follow-up time for both groups. Group A had a lower urinary symptom score than group B at week III and week IV (all p-value < 0.001). In addition, group A had a lower pain score, general condition, work activity, and other complaints than group B at week II, week III, and week IV (all p-value <0.001). The sexual activity score is comparable between the group, except in week I.

**Conclusion:**

according to our results, we suggest tadalafil to minimize stent-related urinary symptoms and improve general health in patients with double J stent.

## Introduction

A ureteral stent is a tool to facilitate the flow of urine from the kidney to the bladder that is disturbed due to obstruction. Ureteral stents have an important role in the temporary drainage of the upper urinary tract and are a frequently performed procedure in endourological surgery [1,2]. Currently, it is found that the most ideal and widely used stent design is the double J stent (DJ stent) design [3]. Complaints related to stents are also known as Stent Related Symptoms (SRSs).

Given the large number of patient complaints, a questionnaire system was developed to evaluate morbidity and side effects after DJ stent placement. The Ureteral Stent Symptoms Questionnaire (USSQ) is the gold standard questionnaire to evaluate the morbidity and side effects of post-DJ stent placement. This questionnaire evaluated complaints that asked about urinary complaints, pain degrees, general health status, work activities, sexual activities, and accompanying complaints that cause discomfort on the subject [4,5].

Along with technological advances and modifications of ureteral stents that continue to be developed to date, various studies to overcome the side effects associated with symptoms after DJ stent placement include modification of stent design, medication, stent position, stent coating, and intravesical therapy [1,6,7]. Several pharmacological agents are also being studied to overcome these side effects. Tadalafil is a selective inhibitor of cyclic guanosine monophosphate (cGMP)-specific phosphodiesterase type 5 (PDE5), which can relax the ureters [8]. Solifenacin is a competitive muscarinic receptor antagonist drug that blocks acetylcholine from binding to M-3 muscarinic receptors in the bladder detrusor muscle, thus preventing bladder contractions. Mirabegrone is a selective agonist for beta-3 adrenergic receptors. Beta-3 adrenoceptors are predominant beta receptors found in detrusor smooth muscle cells, and their stimulation will cause detrusor muscle relaxation [9].

Several previous studies have shown the efficacy of these three drugs in improving SRSs, either single or in combination. Side effects obtained from using this drug are also minimal and well-tolerated [10-13]. However, studies comparing the benefits of SRS improvement obtained from combination therapy and single therapy still need to be completed. Bhattar *et al*.found that 8 mg silodosin and 10 mg solifenacin were superior to 5 mg tadalafil [10]. In another study, tadalafil 10 mg was superior to a placebo [11]. In addition, tadalafil 5 mg is superior to tamsulosin 0.4 mg [12]. Mirabegron 50 mg/day was found to improve SRSs through improvement in international prostate symptom score (IPSS), Overactive Bladder (OAB) symptom score, urinary bother, and visual analog scale for two weeks of treatment [13]. According to inadequate conclusion form previous study, this study aims to compare the efficacy of tadalafil vs a combination of mirabegron and solifenacin in the alleviation of stent-related symptoms in patients with double J stent by a validated questionnaire.

## Methods

**Trial design:** a randomized, double-blind, controlled trial was carried out in the Department of Surgery at Wahidin Sudirohusodo Hospital, University Hospital of Hasanuddin University, Akademis Hospital, and Ibnu Sina Hospital in Makassar City, Indonesia. The trial period was six months, from July 2020 to December 2020.

**Participants:** all patients undergoing unilateral routine DJ stenting after an endourological surgery were enrolled in the study for evaluation. The inclusion criteria are patients with endoscopic indications for insertion of a DJ stent diagnosed with ureteral stones <10 mm (with or without dilatation of the pelvis, calyces, ureter), ureteral stenosis and/or kidney stones who underwent shockwave lithotripsy (ESWL), patients who underwent the first installation of a DJ stent, and unilateral DJ stent complained of SRSs on the seventh postoperative day. The exclusion criteria are patients with a history of malignancy in the urinary tract, previous prostate disease, previous sexual dysfunction, previous urinary tract infection (UTI), pregnancy, chronic medical condition (diabetes mellitus, cardiovascular disease, and hypertension), previous or currently undergoing radiation therapy/hormonal therapy and/or minor pelvic surgery procedures, ureteral reconstruction surgery, history of alcoholism, history of stroke, Alzheimer's, central nervous system trauma, comorbid bladder stones, urethral diverticula in women, and hypersensitivity to drugs such as tadalafil, mirabegron, or solifenacin.

**Interventions:** written informed consent regarding the administration and side effects of therapy was signed by the patient. The baseline characteristics were recorded including age, height, weight, and body mass index (BMI). The additional investigation, including urea/creatinine examination, urinalysis, ultrasonography, abdominal radiograph, and computed tomography (CT) scan urography was performed on the patient before surgery. The size of the largest stone from the patient is used as a benchmark to record the size of the patient's stone.

All patients who underwent routine post-endurological DJ stent insertion (unilateral percutaneous nephrolithotomy (PCNL) or ureteroscopic lithotripsy (URSL)) were included in the study evaluation. The DJ stent used with a type of polyurethane double loop (RocaJJ Soft-ECO Kit, Monaco, Europe) and size of 4.7 Fr was used in all patients with the length of the stent compared to the patient's height.

**Figure 1:**
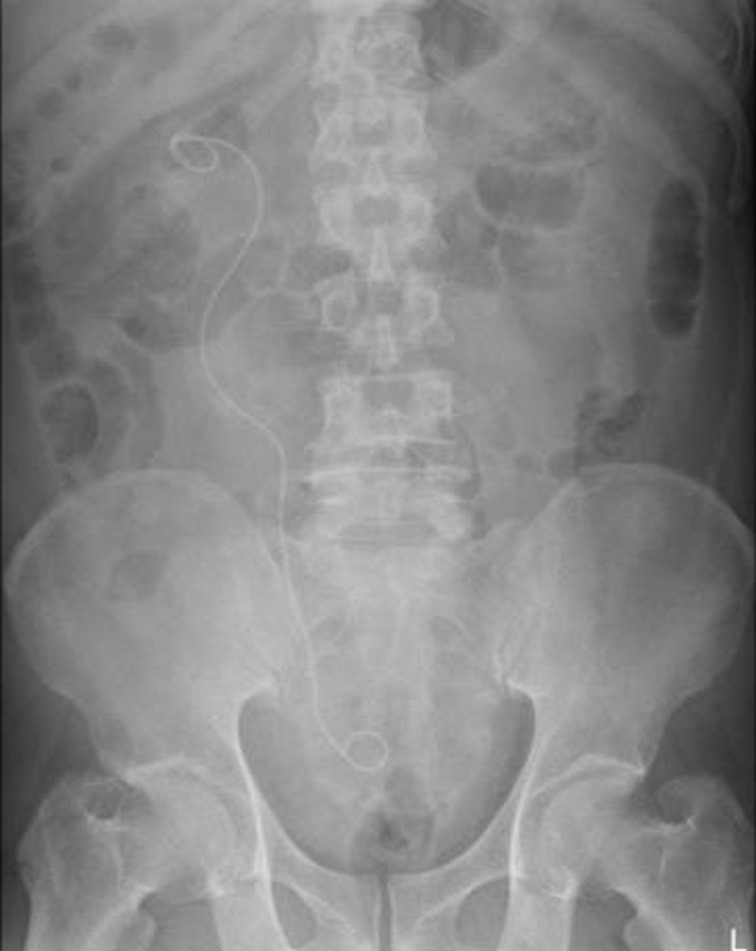
representative image of abdiminal radiograph after successful DJ stent insertion

An abdominal radiograph was taken postoperatively to see the remaining stone fragments and the position of the DJ stent. Figure 1 shows a representative abdominal radiograph image of successful DJ stent implantation. The Foley catheter was removed on the first postoperative day in all patients. The patient was treated as an outpatient after the third postoperative day with oral antibiotics (cefadroxil 500 mg twice daily) for 7 days. The patient was given the same analgesic (paracetamol 500 mg every 8 hours/24 hours and if needed can be given 6 hours/24 hours). The total number of analgesics consumed by the patient will be recorded in the last questionnaire filling.

After determining the sample with exclusion and inclusion criteria, the patient will be divided into 2 groups of drug administration. Group A was given tadalafil 10 mg once daily for 1 month and group B was given a combination of mirabegrone 50 mg once daily and solifenacine 5 mg once daily for 1 month.

In this study, we did not use the placebo group because there have been many studies comparing the placebo group and the effectiveness of the two drugs, so we took only 2 groups to directly compare the effectiveness of the two drugs. Giving a placebo in this case (the group that was not given the drug) is something that should not be done, considering that the patient has complaints that must be treated so that it does not interfere with the patient’s quality of life.

**Outcomes:** the administration of tadalafil 10 mg/day was compared with the combination of mirabegron 25 mg/day and solifenacin 5 mg/day, starting on the seventh day after the insertion of the DJ stent. The patient will be given the USSQ questionnaire format that will measure the presence or absence of complaints of side effects felt by the patient for 7 days after the installation of the DJ stent. Filling out the USSQ questionnaire started on the seventh day after the insertion of the DJ stent, then it was measured every 7 days (days 14, 21, and 28) after the insertion of the DJ stent. Filling out the questionnaire can be done by direct interview at the outpatient clinic or by phone. The USSQ include the following: urinary symptom, pain, general health, quality of work, sexual activity, and other symptoms. The patients were told not to take another medicine and should be visited in situations such as fever, severe pain, and discomfort. Consumption of any other drugs, significant complications, or drug cessation were recorded in each visit. The patients were reminded by a physician the day before each visit by phone call.

**Sample size:** the sample size was calculated using the Openepi with a power 1 - β = 0.8, two-tail α = 0.05, and a difference of 2.0 in the mean of USSQ score change at the final follow-up period (week IV). The required sample size was 38 patients with complaints of SRSs after DJ stent insertion (19 samples in each group). Considering a 30% of dropout and failure to consent, 25 participants in each group are needed.

**Randomization and blinding procedure:** subjects were divided into two groups, each group of 25 patients was simply randomly assigned to treatment (1: 1 ratio). The double-blinded method was used to minimize bias. All medicines were placed in the same 2 boxes, which were held by paramedics, so that patients and researchers did not know the allocation of the type of drug given. All patients have been informed about the side effects of the drug.

**Statistical analysis:** the data was coded, entered, and analyzed using the Ms. Excel 2010, and the statistical analysis used was Statistical Package for the Social Science (SPSS Inc; Chicago, IL, USA) software. Quantitative data including the age, body mass index (BMI), and USSQ scores were presented in the form of mean and standard deviation. The independent t-test was conducted to compare the USSQ score at each time of follow-up between the two groups. The paired t-test was conducted to compare the USSQ score between week I and week IV measurements in the two groups. The categorical data was presented in percent and compared between groups using the chi-square test or Fisher exact test. P-value < 0.05 was considered statistically significant. The analyzed data was presented in the form of frequency tables and cross-tabulation tables, graphs, and narratives for interpretation and discussion.

**Ethics:** this study was carried out after was approved by the Health Research Ethics Committee, Faculty of Medicine, Hasanuddin University Makassar (No. 301/UN4.6.4.5.31/PP36/2021). Before being enrolled in the study, participants received information and were required to sign a written agreement. The collected information was private and anonymous. The randomized clinical trial (RCT) registration number was researchregistry7127 on September 6, 2021.

## Results

**Figure 2:**
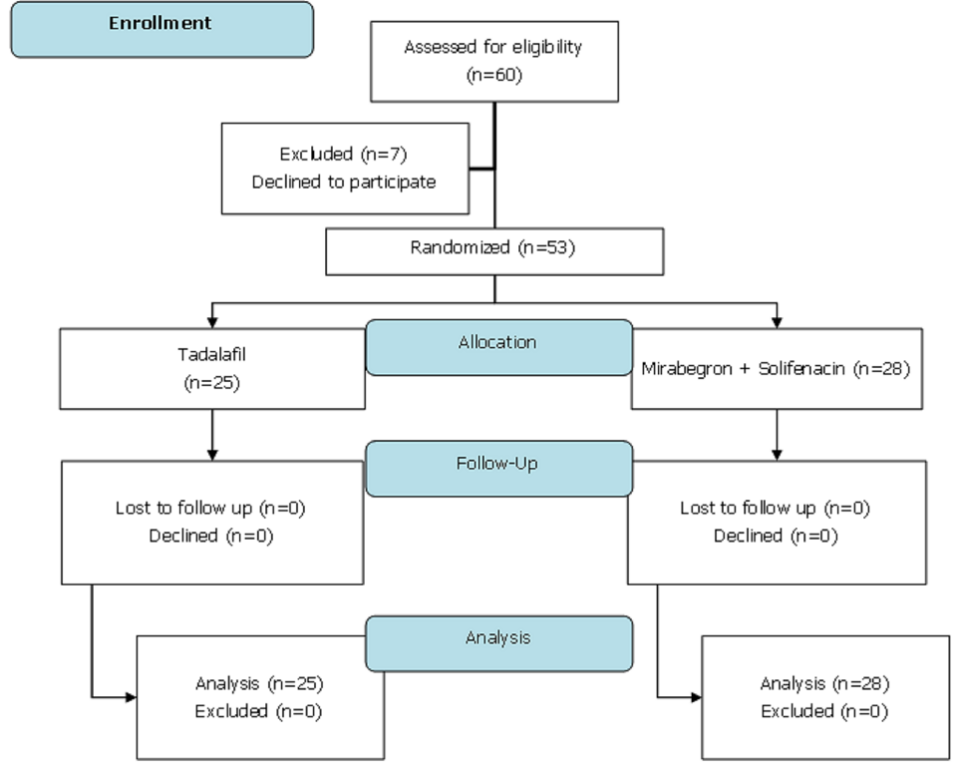
participants flow diagram

**Participants flow:** Figure 2 shows the participant's flow started on assessing for eligibility until follow-up. Between July 1, 2020, and December 31, 2020, 60 participants were screened. Seven participants were excluded for the reason of declining to participate. Fifty-three were randomly allocated (1: 1 ratio) to the tadalafil and mirabegron + solifenacin groups by the principal investigator using sealed opaque envelopes. The tadalafil group (n=25) received the administration of tadalafil 10 mg/day/oral. The mirabegron + solifenacin group (n=28) received the combination of mirabegron 25 mg/day/oral and solifenacin 5 mg/day/oral. The final visit had been completed for all participants at week IV without any loss to follow-up and discontinued participants.

**Baseline data:** Table 1 shows the baseline characteristics of study participants. In the tadalafil group, most of the participants were 26-45 years old (26.42%), male (33.97%), and received right DJ stent insertion (52.00%). In the mirabegron + solifenacin group, most of the participants were 26-45 years old (26.42%), male (39.63%), and received right DJ stent insertion (60.74%). The BMI in the tadalafil group was higher than in mirabegron + solifenacin group (24.86 ± 4.30 kg/m^2^vs 22.83 ± 3.47 kg/m^2^, p=0,048). However, both groups had comparable baseline characteristics in other parameters (all p-value > 0.05).

**Table 1:** baseline characteristics

Variable	Tadalafil (n=25)	Mirabegron + solifenacin (n=28)	p-value
Age (years)	44.64 ± 10.59 (range 24-67)	43.43 ± 10.79 (range 20-60)	0.688*
**Gender**			
Male	18(72.00%)	21(75.00%)	0.805**
Female	7(28.00%)	7(25.00%)	
Height (cm)	164.76 ± 6.09 (range 150-175)	163.35 ± 6.08 (range 150-175)	0.406*
Weight (kg)	66.52 ± 11.91 (range 40-91)	60.92 ± 9.45 (range 42-80)	0.063*
Body mass index (kg/m^2^)	24.43 ± 3.81 (range 17.00-34.60)	22.39 ± 3.51 (range 16.50-31.30)	0.048*
**DJ stent insertion side**			
Right	13(52.00%)	17(60.74%)	0.523**
Left	12(48.00%)	11(39.26%)	
**Indication for DJ stent**			
Uretherolithiasis	14(26.41%)	14(26.41%)	0.906**
Uretheral stenosis	3(5.66%)	4(7.55%)	
Nephrolithiasis	8 (15.09%)	10 (18.87%)	
**Procedures for DJ stent**			
Percutaneus nephrolithotomy	14(26.41%)	14(26.41%)	0.906**
Ureteroscopic lithotripsy	3(5.66%)	4(7.55%)	
ESWL	8 (15.09%)	10 (18.87%)	
**Type of DJ stent**			
Single loop	0(0.00%)	0(0.00%)	1.000^#^
Double loop	25(47.17%)	28(52.83%)	

* independent T-test; **chi-square test, ^#^Fisher exact test; ESWL: extracorporeal shock wave lithotripsy; DJ: double-J

**Figure 3:**
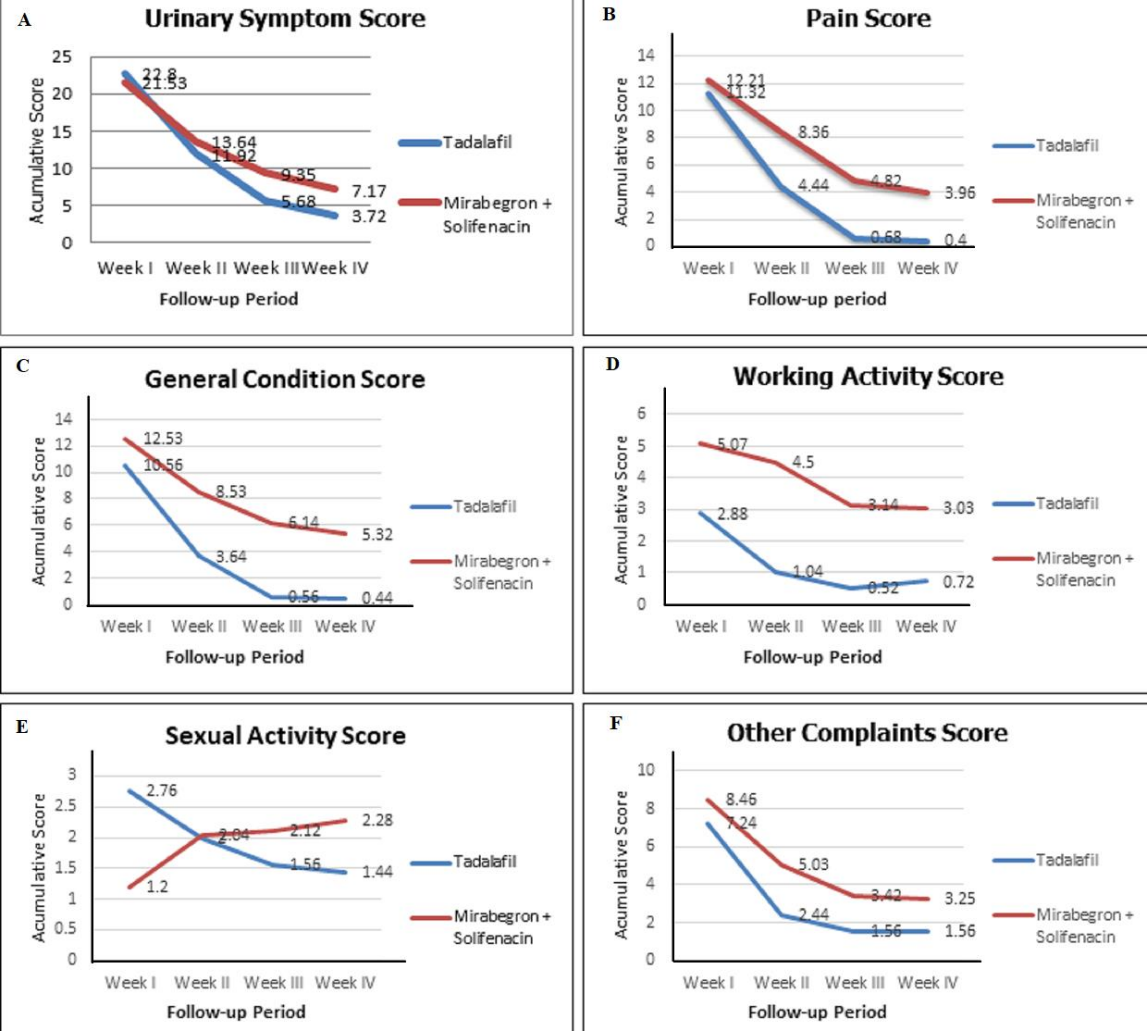
(A,B,C,D,E,F) comparison of Ureteral Stent Symptoms Questionnaire (USSQ) scores in both groups after DJ stent insertion

**Comparison of USSQ scores in both groups after DJ stent insertion:** each subject was included in two groups of observations according to effectiveness comparisons between the two observed pharmacotherapy (Table 2, Figure 3). Differences in USSQ scores in patients after DJ stent placement were divided into differences in urinary symptom scores (US), pain scores (P), general condition (GC), work activity (WA), sexual activity (S), and other complaints (OC).

**Table 2:** comparison of the Ureteral Stent Symptoms Questionnaire (USSQ) scores in both groups after DJ stent insertion

Observation time	Mean score ± SD		p-value*
	Tadalafil (n=25)	Mirabegron + solifenacin (n=28)	
**Urinary symptom (US) score difference**			
Week I	22.80±4.49	21.53 ± 5.79	0.383
Week II	11.92±3.77	13.64±4.63	0.279
Week III	5.68±2.80	9.35±3.71	<0.001
Week IV	3.72±2.09	7.17±3.62	<0.001
Weeks IV vs week I (p-value**)	19.08±4.21 (0.000)	14.36±6.29 (0.000)	
**Pain (P) score difference**			
Week I	11.32±4.05	12.21±4.58	0.458
Week II	4.44±2.70	8.36±3.54	<0.001
Week III	0.68±1.28	4.82±2.80	<0.001
Week IV	0.40±1.04	3.96±2.78	<0.001
Weeks IV vs week I (p-value**)	10.92±3.89 (0.000)	8.25±4.73 (0.000)	
**General condition (GC) score difference**			
Week I	10.56±4.35	12.53±3.81	0.084
Week II	3.64±2.46	8.53±3.71	<0.001
Week III	0.56±1.15	6.14±3.96	<0.001
Week IV	0.44±0.91	5.32±3.22	<0.001
Weeks IV vs week I (p-value**)	10.12±4.19 (0.000)	7.21±3.41(0.000)	
**Work activity (WA) score difference**			
Week I	2.88±3.14	5.07±5.12	0.195
Week II	1.04±1.45	4.50±3.76	0.001
Week III	0.52±1.44	3.14±2.60	<0.001
Week IV	0.72±1.54	3.03±2.51	<0.001
Weeks IV vs week I (p-value**)	2.16±2.56 (0.000)	2.03±3.99 (0.012)	
**Sexual activity (S) score difference**			
Week I	2.76±1.78	1.20±2.02	<0.001
Week II	2.00±0.81	2.04±1.76	0.720
Week III	1.56±0.82	2.12±1.33	0.312
Week IV	1.44 ± 0.87	2.28±0.97	0.036
Weeks IV vs week I (p-value**)	1.32±2.06 (0.004)	-0.96±2.17 (0.026)	
**Other complaints (OC) score difference**			
Week I	7.24±1.92	8.46±2.53	0.061
Week II	2.44±1.47	5.03±2.02	<0.001
Week III	1.56±0.65	3.42±1.47	<0.001
Week IV	1.56±0.65	3.25±1.35	<0.001
Weeks IV vs week I (p-value**)	5.68±2.23 (0.000)	5.21±2.42 (0.000)	

* Independent t-test; **Paired t-test

The US score was lower in the tadalafil group than in the mirabegron + solifenacin group at weeks III and IV (p-value < 0.001). There was no significant difference in the first two weeks, but the two groups tested showed clinical improvement in four weeks of therapy (p-value = 0.000). The P score was lower in the tadalafil group than in the mirabegron + solifenacin group at weeks II, III, and IV (p-value < 0.001). There was no significant difference in the first weeks, but the two groups tested showed clinical improvement in four therapy weeks (p-value=0.000).

The GC scores were lower in the tadalafil group than in the mirabegron + solifenacin group in the follow-up period. However, the GC scores were significantly lower in the tadalafil group than in the mirabegron + solifenacin group only at weeks II, III, and IV (all have p-value < 0.001). Both groups showed a significant decrease in the GC score, indicating the clinical improvement felt by the patient after undergoing therapy for one month (both p-value=0.000).

The WA scores were significantly lower in the tadalafil group than in the mirabegron + solifenacin group in the entire follow-up period (all p-value < 0.05), except at week I (p-value=0.195). Both groups significantly decreased the WA score at the last follow-up period (p-value <0.05). The S scores were significantly higher in the tadalafil group than in the mirabegron + solifenacin group at week I (p-value<0.001) but significantly lower at week IV (p-value=0.036). However, the S scores had comparable means in both groups at week II and week III (p-value > 0.05). Interestingly, the S scores tend to lower during the follow-up period in the tadalafil group (p-value=0.004). Contrarily, the S scores tend to be higher during the follow-up period in the mirabegron + solifenacin group (p-value=0.026). Some of the samples in this study were sexually inactive, such as not having a partner or old age, and the timing of resumption of sexual activity was uneven, so the researchers could not confirm whether there were other external factors associated with this difference in scores, but the scores were statistically significant.

The OC scores constitute the following item: perception of urinary tract infections, sense to consume additional antibiotics, necessity for medical assistance for catheter care, necessity for hospital admission, and perception of happiness in reinserting the DJ stent. The OC scores were significantly lower in the tadalafil group than in the mirabegron + solifenacin group at weeks II, III, and IV (all p-value < 0.001), except week I (p=0.061). Both groups showed a significant decrease in the OC scores indicating the clinical improvement felt by the patient after undergoing therapy for one month (both p-value=0.000).

## Discussion

This study revealed that male patients comprised more than 70% of the occurrence in both group and the majority (52.84%) in the age of 26-45 years. The findings of this study are comparable with those of Pansota *et al*. investigation of the advantages and risks of inserting DJ stents in Pakistan, which assessed 80 patients who had stents. The study indicates that, with a male-to-female ratio of 2.6: 1, most patients (40.0%) ranged in age from 36 to 50 [14].

Post-DJ-stent patients who experienced SRSs were divided into 2 groups and treated with Tadalafil 10 mg/day or a combination of mirabegron 25 mg/day + solifenacin 5 mg/day. Both groups were then observed and assessed using the USSQ questionnaire. According to the study's findings, the tadalafil therapy group exceeded the mirabegron and solifenacin combination group in treating all SRSs.

The findings of this study suggest that treatment with tadalafil 10 mg/day for three weeks in patients having DJ-stents implanted can lessen complaints of urinary symptoms, pain, complaints of general condition, complaints of working activity, complaints of sexual activity, and other complaints. The most effective treatment for patients following DJ-stent insertion can reduce pain complaints using tadalafil 10 mg/day for three weeks. The findings of this study are consistent with those of Haghro *et al*. who assessed 40 patients who were given tadalafil 10 mg/day and found that it significantly decreased lower urinary tract symptoms, pain, sexual function, and the overall score associated with stents [11]. Similar findings were found by Chauhan *et al*. in a study including 72 patients who received tadalafil 10 mg/day from day 7 to day 21. This treatment significantly decreased pain and improved overall health, performance, and sexuality index ratings. In addition, this treatment can reduce lower urinary tract symptoms and enhance patient quality of life [12].

Tadalafil 10 mg in this study can reduce urinary symptoms by 19.08 ± 4.21 points in three weeks. Similar findings were also reported by Bhattar *et al*. using the USSQ scores. They found that urinary symptoms can reduce by 18.07 ± 8.10 points in three weeks of treatment with tadalafil 5 mg. Other parameters were significantly reduced, including pain, general condition, working activity, sexual activity, and other complaints. However, the improvement in pain scores, general condition scores, and other complaints scores were higher in the present study than the previous results in Bhattar *et al*. study (10.92 ± 3.89 vs 3.83 ± 4.96, 10.12 ± 4.19 vs 6.19 ± 4.72, and 5.68 ± 2.23 vs 1.69 ± 3.17, respectively). The higher dosage in the present study may explain the difference in results between this study. Contrarily, the improvement of the working activity and sexual activity in the present study were lower than in Bhattar *et al*. study (1.32 ± 2.06 vs 4.21 ± 2.31 and 2.16 ± 2.56 vs 8.24 ± 5.23, respectively) [10]. Further study must be done to confirm these discrepancies.

Similar to the efficacy of tadalafil in the present study, the combination of mirabegron 25 mg/day + solifenacin 5 mg/day for three weeks can improve the urinary symptoms, pain, general condition, working activity, and other complaints. However, there was a significant in allegations of sex activity. In a study conducted by Abrams *et al*. who compared the combination of mirebegron and solifenacin with monotherapy (mirabergron alone and solifenacin alone) and placebo in patients suffering from overactive bladder (OAB), found that patients receiving combination therapy had better urinary complaints than the monotherapy and placebo group [15]. Another study conducted by Robinson *et al*. (2017) by collecting data on 435 sites in 42 countries also compared the combination of mirebegron and solifenacin with monotherapy (mirabergron alone and solifenacin alone) and placebo in patients suffering from OAB, obtained superior results under combination therapy compared to monotherapy [16].

Based on previous studies, researchers have not found a study comparing the effectiveness of tadalafil 10 mg/day and the combination of mirabegron 25 mg/day + solifenacin 5 mg/day in patients with SRSs. The limitations of this study were that some of the samples in this study were sexually inactive such as not having a partner, old age, and a partner died, as well as the uneven timing of starting sexual activity so that it affected the results on the USSQ questionnaire and the duration of observation in this study lasted for 4 weeks so that only can assess the short-term effect of drug administration on complaints of SRSs. There are other several limitations such as a small sample size, and no comparison after stent removal. The variations and bias in the USSQ questionnaire's five parts have a logical correlation to each other; for example, the patients who had stent-related urinary symptoms have more chance for sexual dysfunction, and the patients who had urinary symptoms, pain, and inappropriate general health had bad quality and quantity of job.

## Conclusion

Tadalafil and a combination of mirabegron and solifenacin improved the SRSs in three weeks. However, additional benefits can be achieved using tadalafil 10 mg. This may imply the single use of tadalafil 10 mg in overcoming SRSs is more considered than the combination therapy, particularly in patients with drug compliance problems. There is a need for further research on the effectiveness of single groups and combination groups with more varied types and doses of drugs, screening for more specific sample characteristics so that the results of the questionnaire conducted can be more homogeneous, and observations with a larger number of samples and a more extended study period to determine short-term and long-term effects and evaluation in therapy in this study.

### What is known about this topic

- *Modification of stent design, stent position, stent coating, and intravesical therapy have overcome the side effects associated with symptoms after DJ stent placement;*
- *Various medications have overcome the side effects associated with symptoms after DJ stent placement*.

### What this study adds

- *The superiority of tadalafil 10 mg/day compared to mirabegron 25 mg/day and solifenacin 5 mg/day combination in overcoming almost all complaints of Stent Related Symptoms (SRSs);*
- *The effectivity of taladafil 10 mg/day did not differ significantly from mirabegron 25 mg/day and solifenacin 5 mg/day combination in overcoming sexual dysfunction*.
